# Non-AIDS-defining comorbidities impact health related quality of life among older adults living with HIV

**DOI:** 10.3389/fmed.2024.1380731

**Published:** 2024-04-16

**Authors:** Alice Zhabokritsky, Marina Klein, Mona Loutfy, Giovanni Guaraldi, Nisha Andany, Silvia Guillemi, Julian Falutz, Gordon Arbess, Darrell H. S. Tan, Sharon Walmsley

**Affiliations:** ^1^Department of Medicine, University Health Network, University of Toronto, Toronto, ON, Canada; ^2^Department of Medicine, McGill University Health Centre, McGill University, Montréal, QC, Canada; ^3^Department of Medicine, Women's College Research Institute, University of Toronto, Toronto, ON, Canada; ^4^Modena HIV Metabolic Clinic, University of Modena and Reggio Emilia, Modena, Italy; ^5^Department of Medicine, Sunnybrook Health Science Centre, University of Toronto, Toronto, ON, Canada; ^6^BC Centre for Excellence in HIV/AIDS, University of British Columbia, Vancouver, BC, Canada; ^7^Unity Health Toronto, Department of Family and Community Medicine, University of Toronto, Toronto, ON, Canada; ^8^Unity Health Toronto, Department of Medicine, University of Toronto, Toronto, ON, Canada

**Keywords:** HIV, multimorbidity, non-communicable diseases, quality of life, stigma

## Abstract

**Introduction:**

The life expectancy of people living with HIV receiving effective combination antiretroviral therapy is approaching that of the general population and non AIDS-defining age-related comorbidities are becoming of greater concern. In order to support healthy aging of this population, we set out to explore the association between multimorbidity (defined as presence of 2 or more non AIDS-defining comorbidities) and quality of life (QoL).

**Methods:**

We performed a cross-sectional analysis using data from the Correlates of Healthy Aging in Geriatric HIV (CHANGE HIV) study, a Canadian cohort of people living with HIV age 65  years and older. Study participants completed two QoL modules, the general QoL and health related QoL (HR-QoL).

**Results:**

433 participants were included in the analysis with a median age of 69  years (interquartile range, IQR 67–72). The median number of comorbidities among study participants was 3 (IQR 2–4), with 78% meeting the definition of multimorbidity. General QoL scores (median 66, IQR 58–76) were lower than HR-QoL scores (median 71, IQR 61–83) and were not associated with multimorbidity after adjusting for age, sex, relationship status, household income, exercise, tobacco smoking history, malnutrition, time since HIV diagnosis, and HIV-related stigma. In contrast, multimorbidity was associated with lower HR-QoL (adjusted β = −4.57, 95% CI −8.86, −0.28) after accounting for the same variables. Several social vulnerabilities (not having a partner, low household income), health behaviours (lower engagement in exercise, smoking), and HIV-related factors (HIV stigma, longer time since HIV diagnosis) were also associated with lower QoL.

**Discussion:**

Overall, our study demonstrated a high burden of multimorbidity among older adults living with HIV in Canada, which has a negative impact on HR-QoL. Interventions aimed at preventing and managing non-AIDS-defining comorbidities should be assessed in people living with HIV to determine whether this can improve their HR-QoL.

## Introduction

People living with HIV who have sustained access to modern combination antiretroviral therapy (cART) are living longer as treatment regimens become more effective, simpler and have fewer toxicities (1, 2). As a result, AIDS-defining illnesses are no longer the primary cause of morbidity and mortality among persons living with HIV (3) and non-AIDS-defining age-related comorbidities, so called non-communicable diseases (NCDs) are becoming of greater concern (4, 5). It has been well recognized that the prevalence of multiple NCDs is greater among people living with HIV, compared to the general population (5–8). Several factors contribute to the increased burden of both individual comorbidities and multimorbidity (≥2 comorbidities) (9, 10) which can be accentuated by modifiable lifestyle and behavioural factors. These factors include persistent immune activation and chronic inflammation despite adequate control of viral replication, co-infections including cytomegalovirus, microbial translocation across the gut and exposure to components of cART itself (10–14).

An association between NCDs and quality of life (QoL) is well known (15, 16). As life expectancy in people living with HIV approaches that of the general population, it is important to sustain and improve well-being during this elongated lifespan (16). Indeed, many have called for the ‘fourth 90’—an aim of health-related QoL (HR-QoL) and healthy aging with HIV to be included as a critical priority for the UNAIDS 90–90-90 targets (16–19). HR-QoL is a multidimensional concept which examines the impact of health status on an individual’s sense of overall function and well-being (20). It is a component of general QoL, which has been defined by the World Health Organization as “an individual’s perception of their position in life in the context of the culture and value systems in which they live and in relation to their goals, expectations, standards and concerns” (21). Popping and colleagues were the first to demonstrate significant progress towards the ‘fourth 90’ among people living with HIV ≥ 18 years of age in the Netherlands and England and found HR-QoL to be highly comparable to the respective general populations (22).

Numerous prior studies have identified lower HR-QoL among cohorts of people living with HIV compared to those in the general population (23–25). Across studies, lower HR-QoL has been associated with multiple factors in people living with HIV, including sociodemographic parameters (i.e., older age, female sex, lower income and less education), HIV-related factors (i.e., longer time since HIV diagnosis, HIV-related stigma), lifestyle (smoking, and substance use disorder) and polypharmacy (26–29). Although NCDs have been shown to have a negative impact on the physical health attributes of HR-QoL in these cohorts, the impact of multimorbidity on general QoL and the non-physical attributes of HR-QoL, especially among older adults living with HIV, is less clear.

To advance this knowledge, we assessed the association between multimorbidity and both general QoL and HR-QOL in an ongoing Canadian cohort of persons living with HIV aged 65 years and older. We hypothesized that multimorbidity would have a negative impact on both measures of QoL.

## Methods

We performed a cross-sectional analysis using baseline data from the Correlates of Healthy Aging in Geriatric HIV (CHANGE HIV) study, an ongoing prospective cohort of people living with HIV age 65 years and older in Canada, established in 2019 (30). Participants were recruited from 7 clinical sites across 3 Canadian provinces (British Columbia, Ontario and Quebec) where they usually access primary and specialty HIV care. The study has received research ethics approval at each of the participating study sites and all participants gave written informed consent.

The study population, instruments and protocol for the main study have been previously described (30). Relative to this analysis, participants completed a sociodemographic interview, a comprehensive review of their medical and HIV-related history (though self-report and medical chart review), and an assessment of lifestyle/behavioural factors. Multimorbidity was defined as presence of ≥2 comorbidities in the same individuals out of the following 20 conditions assessed: hypertension, dyslipidemia, diabetes, coronary artery disease, heart failure, stroke, peripheral arterial disease, cancer, chronic obstructive pulmonary disease, asthma, chronic kidney disease, chronic liver disease, substance use disorder, depression, HIV-associated neurocognitive disorder, Parkinson’s disease, peripheral neuropathy, osteoporosis, arthritis and thrombosis. Nutritional status was evaluated using the Mini Nutritional Assessment (scored from 0–30, with scores of 24 to 30 indicating a normal nutritional status and scores of less than 24 identifying participants who are at risk of or are currently malnourished) (31). HIV-related stigma was measured using the short version of the HIV Stigma Scale (scored from 12 to 48, with higher scores indicating more stigma) (32).

QoL was measured using two Questions on Life Satisfaction questionnaire modules (33). All participants who completed these modules were included in the analysis. The general QoL module assesses 8 domains of life over the preceding 4 weeks, including: “friends/acquaintances”, “leisure time/hobbies”, “health”, “income/financial security”, “occupation/work”, “living conditions”, “family/children” and “relationship with partner/sexual life”. The HR-QoL module assesses 8 health domains over the preceding 4 weeks, including: “physical condition/fitness”, “ability to relax/inner peace”, “energy/enjoyment of life”, “ability to get around”, “vision and hearing”, “freedom from anxiety”, “freedom from discomfort and pain” and “independence from help/care”. For each module, participants first rate how important each of these domains are to them on a scale from 0 to 4 (from not important to extremely important). Next, they rate their own degree of satisfaction with each of those domains on a scale from 0 to 4 (from dissatisfied to very satisfied). For each item, a weighted satisfaction score is calculated using the following formula: importance rating × [(2 × satisfaction rating) – 3] and a summary score for each module is generated (ranging from −96 to +160). The scores then undergo linear transformation to a 0–100 score, where higher scores indicate better QoL (33).

Additionally, to obtain a global rating, participants were asked: “How satisfied are you with your life altogether, if you consider all aspects together?” with response options ranging from dissatisfied to very satisfied on a 5-point Likert scale (adapted from 7th wave of the World Values Survey) (34).

Assuming 75% of people living with HIV age 65 and older have multimorbidity, at an alpha of 0.05, power of 80% and 0.35 effect size, a total sample size of *n* = 343 was required to detect a statistically significant difference in QoL scores using a t-test calculation.

Demographic and clinical variables at cohort entry were summarized using medians and interquartile ranges (IQR) for continuous variables, and counts and frequencies for categorical variables. Mean importance rating for each of the general QoL and HR-QoL domains was summarized according to age category (65–69, 70–74 and ≥ 75 years). Linear regression models were used to evaluate the relationship between the primary exposure (multimorbidity) and the two outcomes of interest (general QoL and HR-QoL). The following variables were selected *a priori* as they may enhance (or mask) the association between multimorbidity and general QoL or HR-QoL: age (in 10-year increments), sex (male vs. female), relationship status (in a relationship vs. unpartnered), household income [dichotomized at the $20,000 per year mark to reflect deep poverty line in Canada (35)], exercise (ordinal variable with 5 levels, ranging from 30 min per day more than once daily to once weekly or less), tobacco smoking history (no smoking history vs. current/past smoking), malnutrition (normal nutritional status vs. at risk of or currently malnourished), time since HIV diagnosis (in 10-year increments), and HIV-related stigma (continuous variable). There was no evidence of collinearity between the selected variables.

## Results

A total of 433 participants were included in the analysis and their characteristics are summarized in Table 1. Median age of participants at time of study enrollment was 69 years (IQR 67–72), 92% male and 76% white. Majority did not have a partner (65%) and were living alone (56%). While most participants had post-secondary education (73%), nearly 1 in 5 were living on a gross household income of less than $20,000 (18%). All participants were on antiretroviral therapy and the viral load was <200 copies/mL in 99.5%. The majority of individuals had evidence of a robust immunologic response to therapy with a current median CD4 cell count of 557 cells/mm^3^ (IQR 409–740). The median number of comorbidities among study participants was 3 (IQR 2–4), with 78% meeting the definition of multimorbidity (≥2 comorbidities) and 53% for severe multimorbidity (≥3 comorbidities). The most commonly encountered comorbidities were dyslipidemia (51%), hypertension (44%), cancer (29%), diabetes (24%), arthritis (22%), peripheral neuropathy (18%), coronary artery disease (17%), depression (15%), osteoporosis (15%) and chronic liver disease (14%).

**Table 1:** Characteristics of CHANGE HIV study participants at cohort entry (*n* = 433).

Characteristic	Median (IQR) or *n* (%)
Age, years – median (IQR)	69 (67–72)
Male sex – *n* (%)	398 (92%)
Race – *n* (%)	
White	328 (76%)
Non-white (self-identified as Indigenous, Asian, Black, Hispanic or other)	104 (24%)
Relationship status – *n* (%)	
In relationship (married, common law, steady partner)	152 (35%)
Unpartnered (single, separated, divorced, widowed)	281 (65%)
Living alone – *n* (%)	241 (56%)
Post-secondary education or higher – *n* (%)	314 (73%)
Household income above $20,000 – *n* (%)	349 (82%)
Exercise, 30 min – *n* (%)	
More than once daily	34 (8%)
Daily	152 (36%)
4–6 times per week	91 (21%)
2–3 times per week	84 (20%)
Once a week or less	66 (15%)
At risk of or malnourished nutritional status – *n* (%)	227 (64%)
Current or past smoking status – *n* (%)	258 (60%)
Alcohol use – *n* (%)	
None	104 (24%)
Occasional	174 (40%)
Weekends only	12 (3%)
2–3 drinks per week	72 (17%)
Daily	70 (16%)
Time since HIV diagnosis, years – median (IQR)	26 (19–31)
Nadir CD4 count, cells/mm^3^ – median (IQR)	200 (100–335)
Current CD4 count, cells/mm^3^ – median (IQR)	557 (409–740)
Number of comorbidities – median (IQR)	3 (2–4)
Multimorbidity – *n* (%)	329 (78%)
Stigma score – median (IQR)	25 (20–29)

Using the global rating, a total of 372 participants (86%) reported being moderately to very satisfied with their life.

**Figure 1:**
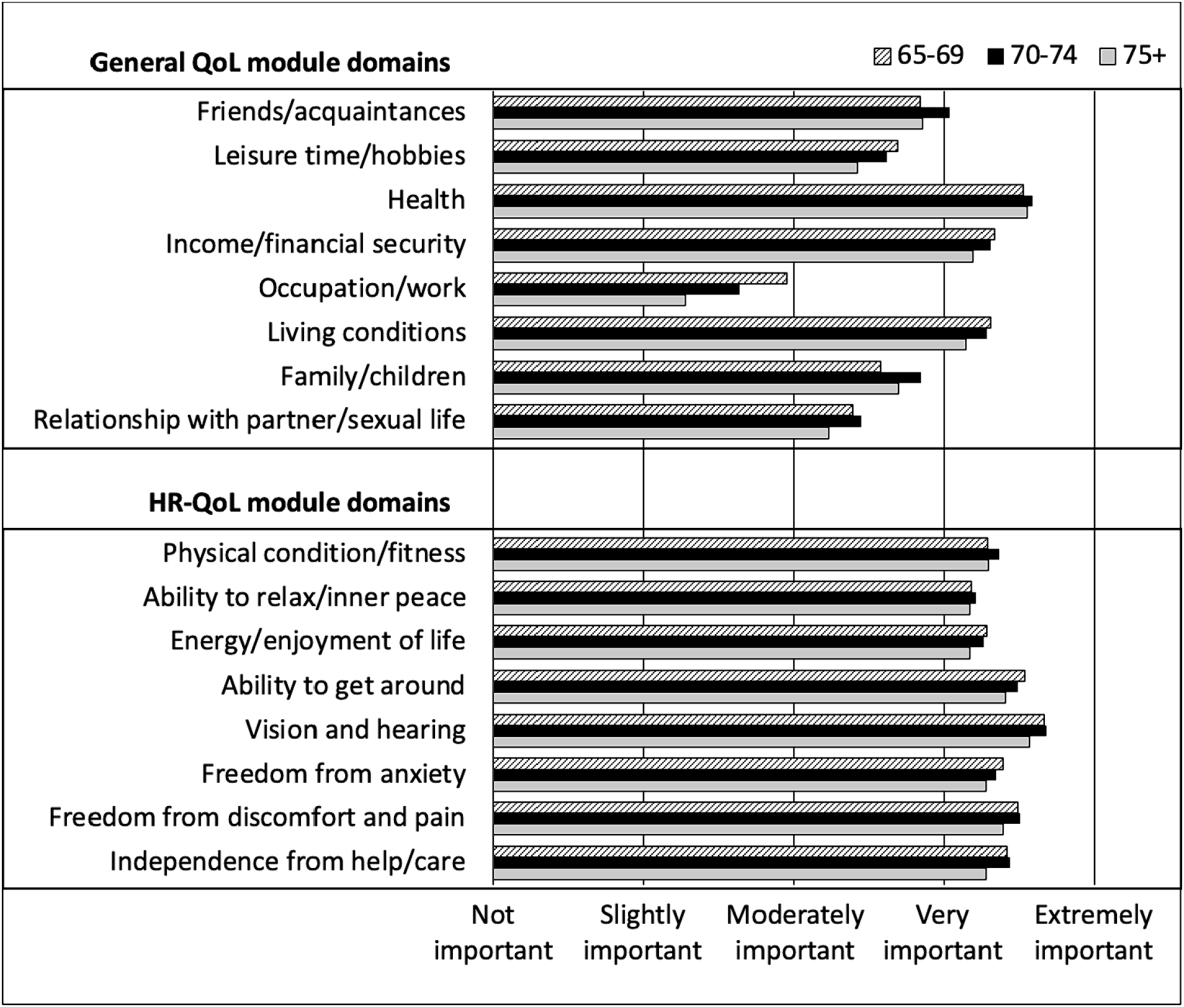
Rating of general QoL and HR-QoL domains importance by CHANGE HIV participants, according to age category.

Looking at the specific QoL modules, on average, participants rated all domains of HR-QoL as very to extremely important, regardless of their age (Figure 1). In contrast, only 3 domains of the general QoL module were rated as very to extremely important (health, income/financial security and living conditions), regardless of age. Occupation/work had the lowest rating of importance among participants (slightly to moderately important and the degree of importance tended to decrease with age) followed by relationship with partner and sexual life, which had less variability according to age.

The median general QoL score among study participants was 66 (IQR 58–76). In univariable analysis, multimorbidity was associated with lower general QoL (β = −3.56, 95% CI −7.00, −0.13) (Table 2). However, in multivariable analysis, multimorbidity was not associated with general QoL scores, after adjusting for age, sex, relationship status, household income, exercise, tobacco smoking history, malnutrition, time since HIV diagnosis, and HIV-related stigma (Table 2). Not being in a relationship (adjusted β = −5.70, 95% CI −8.81, −2.59), having a household income of less than $20,000 (adjusted β = −6.66, 95% CI −10.54, −2.78), exercising less (adjusted β = −1.73, 95% CI −2.89, −0.57), and having a higher degree of stigma (adjusted β = −6.74 per 10-point increase in score, 95% CI −8.94, −4.55) were associated with lower general QoL scores.

**Table 2:** Univariable and multivariable analysis for the outcome of interest of General QoL.

Characteristic	Beta coefficient (95% CI)	Adjusted beta coefficient (95% CI)
Multimorbidity (ref = fewer than 2 comorbidities)	−3.56 (−7.00, −0.13)	−1.85 (−5.33, 1.63)
Age (per 10-year increment)	2.49 (−0.72, 5.70)	0.57 (−2.77, 3.91)
Female (ref = male)	−4.85 (−10.0, 0.29)	−3.85 (−9.63, 1.93)
Not in a relationship (ref = in relationship)	−9.04 (−11.88, −6.20)	−5.70 (−8.81, −2.59)
Household income (ref = above $20,000)	−11.64 (−15.16, −8.12)	−6.66 (−10.54, −2.78)
Exercise (decreasing frequency)	−2.25 (−3.39, −1.10)	−1.73 (−2.89, −0.57)
Smoking (ref = non-smoker)	−3.82 (−6.71, −0.94)	−2.50 (−5.47, 0.46)
At risk of/malnourished (ref = normal nutritional status)	−4.11 (−7.01, −1.21)	−2.25 (−5.20, 0.70)
Time since HIV diagnosis (per 10-year increment)	0.45 (−1.22, 2.11)	−0.26 (−2.01, 1.49)
HIV-related stigma (per 10-point increase in score)	−7.56 (−9.81, −5.32)	−6.74 (−8.94, −4.55)

CI, confidence interval.

The median HR-QoL score was 71 (IQR 61–83). In univariable analysis, multimorbidity was associated with lower HR-QoL (β = −5.73, 95% CI −9.83, −1.64) (Table 3). In multivariable analysis, multimorbidity was still associated with HR-QoL scores (adjusted β = −4.57, 95% CI −8.86, −0.28), after adjusting for age, sex, relationship status, household income, exercise, tobacco smoking history, malnutrition, time since HIV diagnosis, and HIV-related stigma (Table 3). Additionally, having a household income of less than $20,000 (adjusted β = −5.00, 95% CI −9.78, −0.21), exercising less (adjusted β = −2.09, 95% CI −3.52, −0.65), being a current or past smoker (adjusted β = −5.10, 95% CI −8.74, −1.46), longer time since HIV diagnosis (adjusted β = −2.91 per 10-year increment, 95% CI −5.06, −0.75) and having a higher degree of stigma (adjusted β = −8.38 per 10-point increase in score, 95% CI -11.06, −5.69) were associated with lower HR-QoL scores.

**Table 3:** Univariable and multivariable analysis for the outcome of interest of HR-QoL.

Characteristic	Beta coefficient (95% CI)	Adjusted beta coefficient (95% CI)
Multimorbidity (ref = fewer than 2 comorbidities)	−5.73 (−9.83, −1.64)	−4.57 (−8.86, −0.28)
Age (per 10-year increment)	1.00 (−2.78, 4.78)	−1.68 (−5.74, 2.38)
Female (ref = male)	−2.57 (−8.72, 3.58)	−5.43 (−12.47, 1.62)
Not in a relationship (ref = in relationship)	−2.93 (−6.47, 0.60)	0.68 (−3.15, 4.51)
Household income (ref = above $20,000)	−9.70 (−14.04, −5.36)	−5.00 (−9.78, −0.21)
Exercise (decreasing frequency)	−2.52 (−3.90, −1.14)	−2.09 (−3.52, −0.65)
Smoking (ref = non-smoker)	−6.40 (−9.83, −2.97)	−5.10 (−8.74, −1.46)
At risk of/malnourished (ref = normal nutritional status)	−5.56 (−9.05, −2.07)	−2.48 (−6.11, 1.15)
Time since HIV diagnosis (per 10-year increment)	−1.00 (−3.00, 1.00)	−2.91 (−5.06, −0.75)
HIV-related stigma (per 10-point increase in score)	−7.87 (−10.57, −5.17)	−8.38 (−11.06, −5.69)

CI, confidence interval.

## Discussion

In our cohort of older adults (≥ 65 years of age) living with HIV in Canada, the vast majority (86%) of participants reported being moderately to very satisfied with their life when responding to a question on global life satisfaction. However, the median general QoL and HR-QoL scores on validated scales were 66 (IQR 58–76) and 71 (IQR 61–83) respectively, suggesting there is still work to be done towards achieving the “fourth 90” for persons aging with HIV (16).

Our main hypothesis for this analysis, was that multimorbidity would impact the two outcomes of general QoL and HR-QoL. As anticipated, we identified a high burden (78%) of multi-morbidity among our older study participants. While multi-morbidity was associated with lower general QoL and HR-QoL in univariable analyses, after adjusting for potential confounders, multimorbidity only remained associated with lower HR-QoL. This negative impact on HR-QoL could be direct by relation to symptoms associated with specific comorbidities such as pain, immobility, fatigue, or anxiety, or related to treatments used to manage NCDs. Although individuals with multimorbidity tended to have lower general QoL, our findings suggest that this association is more likely indirect. For example, not being in a relationship and having a low household income were independently associated with lower general QoL. Physical and mental disability consequent to NCDs may impact an individual’s ability to develop and maintain social connections, attain adequate housing and financial security, thus contributing to lower general QoL.

Study participants on average rated aspects of HR-QoL as more important than general QoL. Despite high rates of comorbidity and multimorbidity in our study population, the majority of respondents were moderately-very satisfied with their life suggesting that either the comorbidities were well managed or that they had developed effective coping strategies. Therefore, preventing and managing multimorbidity as a way to improve HR-QoL may be more valuable to people living with HIV than measures aimed at improving general QoL.

Our findings expand the previous evidence for the relationship between NCDs and QoL among people living with HIV by focusing on older adults living with HIV (≥65 years) and examining both HR-QoL and general QoL. Previous studies have generally focused on HR-QoL in younger populations (26–28). A study out of the UCSD HIV Neurobehavioral Research Program, demonstrated that a higher comorbidity burden was associated with lower HR-QoL among “younger” (≤40 years, median age 31 years, *n* = 50) and “older” (≥50 years, median age 57 years, *n* = 91) persons living with HIV (27). In the French ANRS CO3 AQUIVIH-NA cohort of 965 people living with HIV age 18 years and older, presence of severe multimorbidity (≥3 comorbidities) was associated with lower HR-QoL (26). In the Dutch AGEhIV cohort study, of 541 people living with HIV with a median age of 53 years (IQR 48–60) a higher number of comorbidities (out of 10 assessed) was associated with worse physical HR-QoL domains but not mental HR-QoL domains (28).

Similar to other studies, we found that stigma continues to have a major impact on QoL in older age, despite advances in HIV therapy (36). HIV related stigma is a major driver of health inequity and intersects with other forms of stigma and discrimination related to race, sexual orientation and gender identity, age, disability, and poverty (37, 38). HIV related stigma can adversely impact both physical and mental health outcomes and can introduce barriers to engagement in care, including management of NCDs (39–41). Indeed, intersectional stigma has been shown to have an association with worse health behaviors and outcomes across multiple studies, including lower medication adherence, delayed or avoided care, substance use, and concealment of stigmatized behaviours and conditions in personal and healthcare settings (i.e., HIV status, sex work, hepatitis C co-infection) (42–47). As such, ongoing efforts to develop and implement effective stigma reduction interventions are crucial to improving QoL among people living with HIV across the lifespan (48).

Additionally, we found an association between longer time since HIV diagnosis and lower HR-QoL. In our cohort of older adults living with HIV, this likely reflects experiences of long-term survivors who were diagnosed in the pre-cART era, many of whom endured delays in accessing effective treatment resulting in profound immunodeficiency, were exposed to long-term toxicities related to early antiretroviral drugs which could impact pain or mobility and experienced multiple traumatizing adverse life events (49, 50). As we strive for earlier detection and initiation of effective treatment for HIV, along with prevention and improved management of NCDs, it will be important to determine whether this association can be reversed.

Integrated health care models with the ability to address the multimorbidity related needs of persons aging with HIV may lead to better health and quality of life outcomes. We must better prevent, screen and manage comorbidities and their impact on quality of life in an individualized manner, while recognizing that some of these processes may be more chronic while others episodic in nature. The incorporation of patient reported outcomes in research and in care, may better reflect the individual perception of their health, quality of life and well-being.

Our study has several limitations. The cross-sectional nature of our analysis prevents us from making any causal inferences. Additionally, we assessed comorbidity burden across 20 common comorbid conditions, however, disease severity and impact of treatment were not taken into consideration in this analysis. The rate of multimorbidity was high and we were unable to assess the impact of the individual comorbidities on QoL. Study participants were recruited into the cohort from urban clinic sites, where they receive HIV care and have access to multidisciplinary teams and various health resources. This may contribute to earlier diagnosis and management of NCDs and better management of associated symptoms, resulting in an underestimated effect of multimorbidity on QoL. Those recruited were also well enough to attend clinic in person, provide informed consent and participate in lengthy study protocols, thereby excluding individuals with significant physical or cognitive impairment. As such, our findings may not be generalizable to individuals with more advanced disease, those not engaged in care or receiving HIV care in different settings, or other populations of older adults living with HIV from different demographic backgrounds.

In conclusion, older adults living with HIV experience a high burden of NCDs, which has a negative impact on HR-QoL. Interventions aimed at preventing and managing NCDs should be assessed in people living with HIV to determine whether this can improve their HR-QoL. Additional targets to improve QoL may include interventions which address HIV-related stigma, facilitate smoking cessation and increase exercise participation. A differential approach may be needed to address the needs of long-term survivors of HIV and those who do not have a partner or live alone, as these individuals tend to experience a lower QoL even when accounting for comorbidity burden.

## Data availability statement

The original contributions presented in the study are included in the article/supplementary material, further inquiries can be directed to the corresponding author.

## Ethics statement

The studies involving humans were approved by University Health Network Research Ethics Board. The studies were conducted in accordance with the local legislation and institutional requirements. The participants provided their written informed consent to participate in this study.

## Author contributions

AZ: Data curation, Formal analysis, Investigation, Methodology, Software, Visualization, Writing – original draft, Writing – review & editing. MK: Conceptualization, Data curation, Investigation, Methodology, Resources, Validation, Writing – review & editing, Funding acquisition, Project administration, Visualization. ML: Writing – review & editing, Conceptualization, Data curation, Funding acquisition, Investigation, Methodology, Project administration, Resources, Validation, Visualization. GG: Conceptualization, Funding acquisition, Methodology, Validation, Writing – review & editing, Visualization. NA: Conceptualization, Data curation, Funding acquisition, Investigation, Methodology, Project administration, Resources, Validation, Visualization, Writing – review & editing. SG: Conceptualization, Data curation, Funding acquisition, Investigation, Methodology, Project administration, Resources, Validation, Visualization, Writing – review & editing. JF: Conceptualization, Data curation, Funding acquisition, Investigation, Methodology, Project administration, Resources, Validation, Visualization, Writing – review & editing. GA: Conceptualization, Data curation, Funding acquisition, Investigation, Methodology, Project administration, Validation, Visualization, Writing – review & editing. SW: Conceptualization, Data curation, Funding acquisition, Investigation, Methodology, Project administration, Resources, Supervision, Validation, Visualization, Writing – review & editing. DT: Conceptualization, Data curation, Funding acquisition, Investigation, Methodology, Project administration, Resources, Validation, Visualization, Writing – review & editing.
